# Cigarette Smoking Associated with Colorectal Cancer Survival: A Nationwide, Population-Based Cohort Study

**DOI:** 10.3390/jcm11040913

**Published:** 2022-02-09

**Authors:** Yu-Min Huang, Po-Li Wei, Chung-Han Ho, Chih-Ching Yeh

**Affiliations:** 1 Department of Surgery, School of Medicine, College of Medicine, Taipei Medical University, Taipei 11031, Taiwan; yuminhuang26@gmail.com (Y.-M.H.); poliwei@tmu.edu.tw (P.-L.W.); 2 Division of General Surgery, Department of Surgery, Taipei Medical University Hospital, Taipei 11031, Taiwan; 3 Division of Colorectal Surgery, Department of Surgery, Taipei Medical University Hospital, Taipei Medical University, Taipei 11031, Taiwan; 4 Department of Medical Research, Chi-Mei Medical Center, Tainan 71004, Taiwan; ho.c.hank@gmail.com; 5 Department of Information Management, Southern Taiwan University of Science and Technology, Tainan 71005, Taiwan; 6 Master Program in Applied Epidemiology, College of Public Health, Taipei Medical University, Taipei 11031, Taiwan; 7 Cancer Center, Wan Fang Hospital, Taipei Medical University, Taipei 11696, Taiwan; 8 School of Public Health, College of Public Health, Taipei Medical University, Taipei 11031, Taiwan; 9 Department of Public Health, College of Public Health, China Medical University, Taichung 40402, Taiwan

**Keywords:** colorectal cancer, cigarette smoking, survival

## Abstract

We investigate whether cigarette smoking is associated with survival in patients with colorectal cancer (CRC) through a nationwide population-based cohort study in Taiwan. The Taiwan Cancer Registry and National Health Insurance Research Database were used to identify data from patients with CRC from 2011 to 2017. Tobacco use was evaluated based on the smoking status, intensity, and duration before cancer diagnosis. A total of 18,816 patients was included. A Kaplan–Meier survival analysis indicated smoking to be significantly associated with the CRC mortality risk (log-rank *p* = 0.0001). A multivariable Cox model indicated that smoking patients had a 1.11-fold higher mortality risk (HR = 1.11, 95% CI = 1.05–1.19) than nonsmoking patients did. This increased risk was also present in patients with CRC who smoked 11–20 cigarettes per day (HR = 1.16; 95% CI = 1.07–1.26) or smoked for >30 years (HR = 1.14; 95% CI = 1.04–1.25). Stratified analyses of sex and cancer subsites indicated that the effects of smoking were higher in male patients and in those with colon cancer. Our results indicate that cigarette smoking is significantly associated with poor survival in patients with CRC. An integrated smoking cessation campaign is warranted to prevent CRC mortality.

## 1. Introduction

Colorectal cancer (CRC) is among the most common cancers and among the leading causes of cancer deaths worldwide [1,2,3,4]. Although it has historically been more prevalent in the West, the incidence rates of CRC have been increasing in East Asian countries [5]. In Taiwan, CRC is one of the most commonly diagnosed cancers [6]. Despite the progress that has been achieved in its diagnosis and treatment, approximately half of patients with CRC die within 5 years of diagnosis [4]. Therefore, further efforts to identify and obviate the risk factors of CRC mortality are required to improve the prognosis of this cancer.

Cigarette smoking is a serious public health concern; it is annually responsible for millions of deaths around the world [7]. Smoking is estimated to be responsible for more than 30% of cancer deaths in the United States each year. Smoking has also been observed to increase the risk of mortality in CRC [8]. The association between smoking and CRC has been demonstrated in many studies [9,10,11,12,13]. Long-term smokers have been reported to have a significantly increased risk of developing CRC than nonsmokers [13,14,15,16]. Studies have reported a 15% to 60% higher risk estimate associated with active smoking [1,17,18]. Although data were insufficient for the association between smoking and CRC to be defined as casual, recent studies have suggested cigarette smoking to be a risk factor for CRC [1,4,13,19,20,21]. Consequently, the American College of Gastroenterology colorectal cancer screening guidelines have highlighted smokers as being at an increased risk [22].

Cigarette smoking may worsen the prognosis of CRC [23,24]. Long-term cigarette smoking has been reported to increase the risk of both overall and disease-specific CRC mortality in men and women [4,9,13,19]. However, findings regarding the influence of smoking on CRC survival have been inconsistent; several studies have also reported no significant association between smoking and CRC mortality [25,26].

Moreover, many of the aforementioned studies on the association between smoking and the risk and prognosis of CRC were conducted in Western countries [5,27]. In the 16 studies included in one meta-analysis, only 1 was conducted in East Asia [28]. Therefore, because of factors such as ethnicity, culture, and lifestyle, the reported findings of this meta-analysis may not be directly applicable to other demographic groups. Another meta-analysis reported that the relative risks (RRs) of CRC among current smokers were significantly different in different geographic areas [1]. In addition, the results of studies from Asian countries have generally been heterogeneous, which further complicates the matter [5]. Evidence regarding the effects of cigarette smoking on the prognosis of CRC remains limited. Therefore, we perform a nationwide population-based cohort study to investigate whether cigarette smoking adversely affects the survival outcomes of Asian patients with CRC.

## 2. Materials and Methods

### 2.1. Data Sources

Data in this study were collected from the Taiwan Cancer Registry (TCR) and Taiwan’s National Health Insurance Research Database (NHIRD). Both of these databases are managed by the Health and Welfare Data Science Center (HWDC) of the Ministry of Health and Welfare. The TCR was established to gather information on individual demographics, cancer stages (AJCC 7th edition), primary sites, histology, and treatment types in patients with cancer to understand the incidence and mortality rates of cancer in Taiwan. The NHIRD was established for research purposes; it contains data from Taiwan’s single-payer insurance system, in which more than 99% of Taiwan’s 23 million citizens are registered. The NHIRD contains registration files and original inpatient and outpatient reimbursement claim data from 1996 to 2017. The datasets of the HWDC are all de-identified forms. This study was conducted in compliance with the Declaration of Helsinki of 1964 and was approved by the Research Ethics Committee of Chi Mei Hospital (IRB no. 10702-E04). The requirement for informed consent was waived by the Research Ethics Committee of Chi Mei Hospital.

### 2.2. Study Population

**Figure 1:**
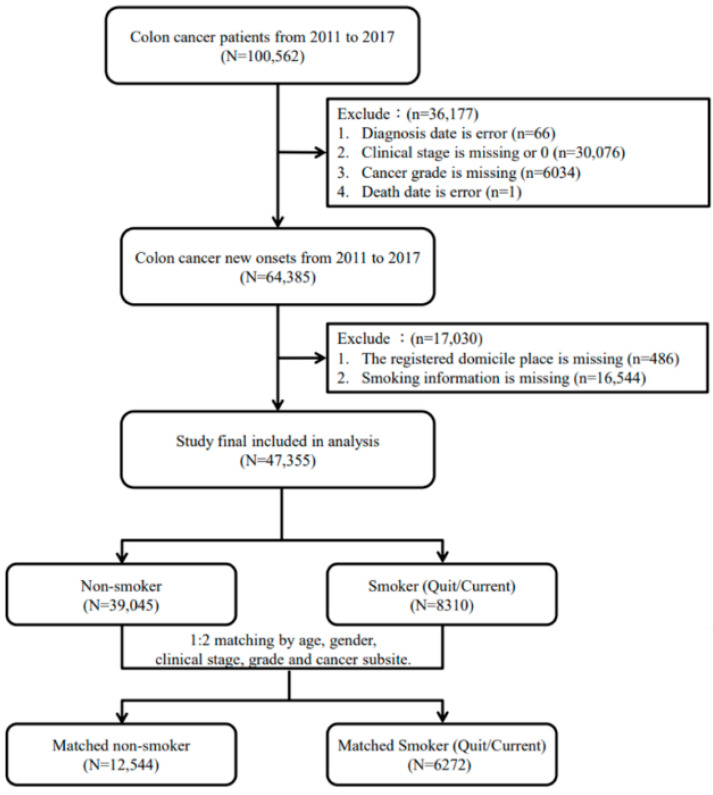
Flowchart of study population selection.

The TCR was used to identify patients with CRC based on the International Classification of Diseases for Oncology, third edition (ICD-O-3); in this study, colon (ICD-O-3: C18), rectosigmoid junction (ICD-O-3: C19), and rectum (ICD-O-3: C20) cancers were included. Because the TCR began recording smoking and drinking behavioral information in 2011, patient data from 2011 to 2017 were selected. Patients with a history of CRC before 2011 were excluded to reduce omitted variable bias. In addition, because the aim of this study was to estimate the association between cigarette smoking and risk of mortality in patients with CRC, included patients were categorized as those with and without a history of prediagnostic smoking. Those with a smoking history included both current and ever smokers, for whom the duration of smoking in years and smoking count per day were included in the analysis. To reduce the potential confounding factors of mortality, including age, gender, clinical stage, grade, and cancer subsite, between patients with smoking and those without, we randomly selected two patients without smoking to match each patient with smoking using propensity score approach. A propensity score matching approach with the nearest-neighbor matching algorithm was used in this study according to SAS macro “*%OneToManyMTCH*”. The flowchart of the study population selection is presented in Figure 1.

### 2.3. Measurements

The major outcome of this study was overall mortality. Mortality was defined using Taiwan’s cause-of-death data. All patients were right censored to date of death or 31 December 2017, whichever came first. The study variables, namely, age at diagnosis, sex, clinical stage, histological grade, and alcohol drinking habit, were all collected from the TCR. Age was divided into groups of <40, 40–49, 50–59, 60–69, and ≥70 years. Charlson comorbidity index (CCI) scores were calculated using patients’ diagnosis records from the NHIRD to represent patients’ comorbidities, which were defined before the date of diagnosis of CRC. To generate the index score, each of the 19 identified medical conditions was scored from 1 to 6.

### 2.4. Statistical Analysis

The frequency was presented as a percentage for categorical variables among the study population. The distribution difference between smoking and nonsmoking groups was compared using Pearson’s chi-square test. The trend of mortality during the study period was plotted using the Kaplan–Meier approach, with a log-rank test for estimating the statistical difference between smoking and nonsmoking patients with CRC. Multivariable Cox proportional regression was constructed to estimate the mortality risk and control for potential confounders by adjusting for age, sex, drinking habit, residence in a remote area, cancer site, cancer clinical stage, cancer grade, and CCI group. Stratified analyses of age, sex, and CRC subsites were also presented. To observe the progress of mortality risk on smoking counts per day and smoking years, the linear trend test was used to estimate the potential trends. All analyses were conducted using SAS statistical software version 9.4 (SAS Institute, Cary, NC, USA). Significance was set at *p*  <  0.05. Kaplan–Meier curves were plotted using STATA (version 12; Stata, College Station, TX, USA).

## 3. Results

### 3.1. Characteristics of Study Population

The baseline characteristics of the matched cohort are presented in Table 1. Of the 18,816 patients with CRC included in this study, 6272 were smokers and 12,544 were not. The smoking group comprised more patients with drinking habits (52.1% vs. 13.8%, *p* < 0.0001). In addition, the mortality rate was significantly higher in the smoking group (30.1% vs. 27.9%, *p* = 0.0012). Otherwise, the two groups were balanced with regard to age, sex, residence in remote area, cancer subsite, clinical stage, tumor grade, and CCI grouping.

### 3.2. Cigarette Smoking and Mortality Risk

**Figure 2:**
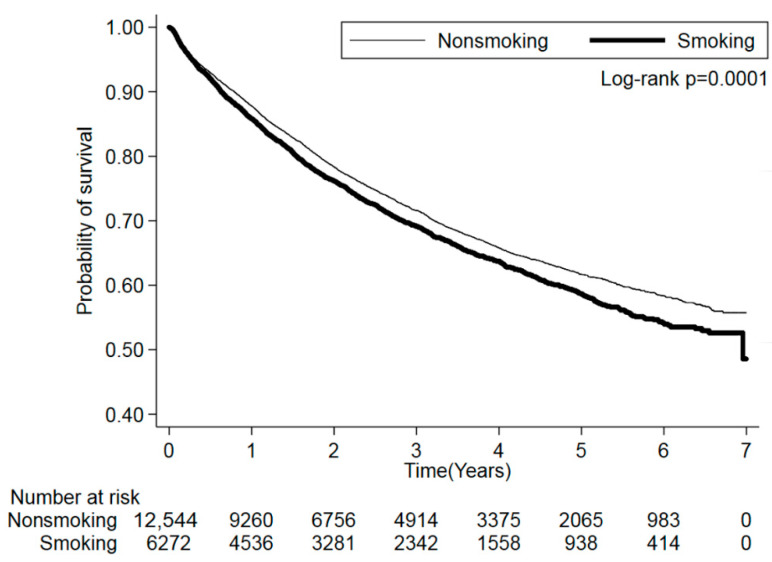
Association of cigarette smoking with mortality risk of colorectal cancer.

As illustrated in Figure 2, a significant difference was found in the mortality risk of CRC between the smoking and nonsmoking groups (log-rank test *p* = 0.0001). The crude data, presented in Table 2, revealed that smoking patients had a 1.11-fold higher mortality risk (95% CI = 1.05–1.19; *p* = 0.0009) than nonsmoking patients did. Regarding the effects of smoking intensity, patients who smoked 11–20 cigarettes per day (HR = 1.17; 95% CI = 1.08–1.27; *p* = 0.0001) and who smoked for more than 10 years (HR = 1.12; 95% CI = 1.02–1.23; *p* = 0.0184 for patients smoking for 11–30 years; HR = 1.15; 95% CI = 1.06–1.26; *p* = 0.0014 for those smoking for >30 years) had a significantly higher mortality risk than nonsmoking patients did. After adjustment for age, sex, alcohol-drinking habit, residence in remote areas, cancer subsites, cancer clinical stage, cancer tumor grade, and CCI score grouping, smoking patients had a 1.10-fold higher mortality risk (95% CI = 1.03–1.18; *p* = 0.0056) than nonsmoking patients did. Patients who smoked 11–20 cigarettes (HR = 1.16; 95% CI = 1.07–1.26; *p* = 0.0006) per day and who smoked for more than 10 years (HR = 1.11; 95% CI = 1.01–1.23; *p* = 0.0356 for patients smoking for 11–30 years; HR = 1.14; 95% CI = 1.04–1.25; *p* = 0.0044 for those smoking for >30 years) had a significantly higher mortality risk than nonsmoking patients did. A significant trend was identified for increased mortality risk due to smoking duration (*p* = 0.0474).

### 3.3. Cigarette Smoking and Mortality Risk Stratified by Sex

The data presented in Table 3 revealed the risk of mortality associated with smoking in patients with CRC stratified by sex. The proportions of male and female smokers were the same (33.3%), but the male smoking population was 9.47 times of the female. Smoking men had a 1.09-fold higher mortality risk (95% CI = 1.02–1.18; *p* = 0.0156) than nonsmoking men did. A further analysis revealed a significantly higher risk of mortality in men who smoked 11–20 cigarettes per day (HR = 1.16; 95% CI = 1.06–1.26; *p* = 0.0011) and who smoked for more than 10 years (HR = 1.11; 95% CI = 1.01–1.24; *p* = 0.0390 for men smoking for 11–30 years; HR = 1.14; 95% CI = 1.04–1.25; *p* = 0.0076 for those smoking for >30 years) than in those who were nonsmokers. For women, a 1.59-fold higher mortality risk was observed only in those who smoked for 1–10 years (95% CI = 1.03–2.45; *p* = 0.0367) compared with nonsmoking women. However, the increase in risk was not significant in the other levels of smoking intensity.

### 3.4. Cigarette Smoking and Mortality Risk Stratified by Cancer Subsite

The risk of mortality associated with smoking with respect to the CRC subsite is presented in Table 4. Smoking patients were associated with a significantly higher mortality risk than nonsmoking patients were for colon (HR = 1.12; 95% CI = 1.03–1.22; *p* = 0.0096) but not rectal cancers (HR = 1.08; 95% CI = 0.95–1.22; *p* = 0.2339). Furthermore, patients with colon cancer who smoked more than 10 cigarettes (HR = 1.15; 95% CI = 1.04–1.28; *p* = 0.0072 for patients smoking 11–20 cigarettes; HR = 1.18; 95% CI = 1.01–1.39; *p* = 0.0376 for those smoking >20 cigarettes) daily and who smoked for more than 10 years (HR = 1.13; 95% CI = 1.00–1.28; *p* = 0.0447 for patients smoking for 11–30 years; HR = 1.18; 95% CI = 1.05–1.32; *p* = 0.0046 for those smoking for >30 years) had a significantly higher mortality risk than nonsmoking patients did. A significant trend was also identified for an increased mortality risk due to smoking intensity (*p* = 0.0463). By contrast, no significant increase in mortality risk was observed in patients with rectal cancer except in those smoking 11–20 cigarettes per day (HR = 1.17; 95% CI = 1.01–1.35; *p* = 0.0337).

### 3.5. Cigarette Smoking and Mortality Risk Stratified by Age

The effects of smoking on the mortality risk were assessed with the stratification of the patients’ ages (Table 5). For patients younger than 60, an increased CRC mortality risk was not associated with smoking, with the exception of those who smoked for more than 30 years (HR = 1.43; 95% CI = 1.10–1.87; *p* = 0.0074). By contrast, smoking significantly increased the risk of mortality in patients with CRC who were older than 60 (HR = 1.12; 95% CI = 1.03–1.21; *p* = 0.0061). Higher risks were observed in such patients who smoked 11–20 cigarettes per day (HR = 1.16; 95% CI = 1.05–1.27; *p* = 0.0034) and who smoked for more than 10 years (HR = 1.15; 95% CI = 1.02–1.30; *p* = 0.0190 for patients who smoked for 11–30 years; HR = 1.12; 95% CI = 1.02–1.24; *p* = 0.0209 for those who smoked for more than 30 years).

## 4. Discussion

Through the combined analysis of data from nationwide health insurance and cancer registries, we demonstrated that cigarette smoking was associated with a significantly increased risk of mortality in patients with CRC. The increased risk was more prominent in patients with higher levels of smoking intensity and duration. This pattern was especially present in men, patients with colon cancer, and patients older than 60. A dose–response effect on the risk of mortality was also observed with smoking duration in the whole population and with smoking amount in patients with colon cancer. Although the increase in the mortality risk was moderate in most of the analyzed categories, its significance merits further consideration to improve the prognosis of CRC.

Despite widespread skepticism towards an association between cigarette smoking and CRC, accumulating evidence has suggested an increased risk of incidence incurred with smoking [5]. In the Iowa Women’s Health Study (IWHS), ever smokers had a moderately increased CRC risk (RR of approximately 1.20) compared with never smokers [11,27]. In the Cancer Prevention Study II Nutrition Cohort, the incidence of CRC was approximately 30% higher in current smokers than in never smokers [18]. Two meta-analyses demonstrated that the pooled RR increased from 15% to 20% in ever smokers compared with never smokers [1,16].

Furthermore, cigarette smoking was associated with an increased risk of mortality in patients with CRC. In the IWHS, ever smokers had an increased risk of overall mortality (RR = 1.31) compared with never smokers, which was similar to observations of CRC incidence [11]. In a previous meta-analysis, current smokers exhibited a significantly higher risk of CRC mortality (RR = 1.58) compared with nonsmokers [16]. In addition to all-cause mortality, disease-specific mortality was affected by current smoking [23,24]. Further evidence of this was presented in a meta-analysis that demonstrated that smokers had a 26% higher risk of all-cause mortality than never smokers did. Notably, 30-day mortality was reported to be higher by between 49% and 100% [4]. Compared with these previous studies, the mortality risk increased only moderately, though significantly, in smokers in our study. Factors such as components in cigarettes or differences in the study population might be implicated.

Our results indicated an increased risk of mortality in patients who smoked more than 10 cigarettes per day and who smoked for more than 10 years. This amount and the duration were much lower than previously reported. In the Chicago Heart Association cohort, a significant association between smoking and an increased CRC mortality was observed only in patients who smoked more than 20 cigarettes per day [19]. Smoking more than 15 cigarettes per day and having a 20-pack-year history were reported by Walter et al. to affect CRC survival [4]. In addition to differences in the characteristics of cigarettes and study populations, the lower threshold of smoking intensity for an increasing CRC mortality risk in our study may be attributable to the numerous events that may increase the detectability of differences in risks.

The mechanisms underlying the association between smoking and CRC mortality are multifold and incompletely understood. Cigarette smoke contains more than 60 carcinogens [7,17,29]. Of them, nicotine and 4-(methylnitrosamino)-1-(3-pyridyl)-1-butanone may enhance metastasis, which is the leading cause of death in patients with CRC, by enhancing cell migration and epithelial–mesenchymal transformation [6,30,31]. Nicotine may also interfere with the antiproliferative and proapoptotic effects of chemotherapeutic agents [4,32,33]. Tobacco smoking may cause a mutation of the GSTM1 gene, resulting in the impaired detoxification of tobacco carcinogens and enhancement of carcinogens’ tumorigenic actions. Smoking may also induce aberrant promoter DNA methylation and silence regulatory genes in tumor progression. Consequent genetic alterations, such as a high microsatellite instability (MSI), the CpG island methylator phenotype, and the BRAF V600E mutation, may impair patient survival [13].

In previous studies, risk factors associated with an increased CRC mortality in smokers included an active smoking status, increased smoking amount or duration, and younger age at initiation [4,9,13,18]. The effects of smoking were also more significant in patients younger than 50 [19,24]. Notably, in our study, smoking patients younger than 60 had a lower risk of mortality. An exception to this was the increased risk in those who smoked for more than 30 years, indicating effects of prolonged smoking duration and younger age at initiation. By contrast, a generally significant association was noted between smoking and an increased risk of mortality in patients older than 60. This contradicted the observations by Colangelo et al. that the association between CRC and smoking mortality was significant only in those younger than 50 [19]. Whether this variance can be explained by other unanalyzed factors, such as genetic alterations, requires further elucidation.

For the effect of sex, Colangelo et al. reported that the risk for CRC mortality was higher for women than for men at the same level of smoking exposure, a phenomenon similar to that observed in patients with lung cancer [19]. However, other studies have reported discordant results. The association between smoking and the risk of CRC mortality was higher in men in a study conducted in Canada [13]; the association was even greater in patients older than 60. Walter et al. and Phipps et al. also reported a greater risk of recurrence or mortality in male smokers [4,24]. In our study, the increased risk in male smokers remained significant and relatively constant at most levels of smoking amount and duration. By contrast, the increases in risk in female smokers were mostly nonsignificant. Although our results may suggest a higher CRC mortality risk in male smokers, the disproportionately low number of female smokers when compared with that of male smokers may attenuate the association in females [4,9,11,18,19,21].

Tumor-related factors associated with an increased risks of recurrence or mortality in smokers include a T3 tumor, one to three affected lymph nodes, nonmetastatic diseases, a mutated KRAS status, and a wild-type BRAF status [4,24]. The effect of MSI remains under debate; some studies have suggested that the associations of smoking with all-cause CRC mortality were higher among patients with microsatellite-stable or MSI-low tumors, whereas others have reported a similar association with MSI-high tumors [4,13,24]. Data regarding genetic analyses of CRC specimens were not available in our database, which precludes the further exploration of the mechanisms underlying the association between cigarette smoking and an increased risk of CRC mortality.

Clinically, cigarette smoking is associated with later stages of CRC at diagnosis, which leads to a poorer prognosis and survival [34]. However, the increased risk persisted in our study despite matching for cancer stage. In addition to a proneoplastic effect, tobacco smoking constitutes a primary risk factor for cardiovascular and pulmonary diseases [32]. Therefore, smoking patients with CRC may incur additional risk or mortality from these causes. A prolonged induction period of more than 35 years is required for smoking to increase the risk of incident CRC [10,11,14,20]. The shorter duration of smoking associated with an increased CRC mortality risk in our study may support the involvement of smoking-induced comorbidities.

Studies have indicated that colon and rectal cancer may have partly different etiologic pathways and should be considered to be two separate entities that differ in susceptibility to carcinogens [11,17]. However, no consensus has been reached regarding whether the risk of incident colon or rectal cancer is more strongly associated with smoking [1,10,11,12,16,17,18,20,21,27,35,36,37,38,39]. Similarly, the association between smoking and CRC subsite mortality has been the topic of debate; several studies have reported that smoking was more significantly associated with a worse survival in patients with colon cancer than in those with rectal cancer [4,13,24]. However, others have reported a similar association between smoking and colon and rectal cancer mortality [9]. In our study, smoking was associated with an increased risk of mortality in patients with colon but not rectal cancers, which implies a higher susceptibility in patients with colon cancer. A significant dose–response relationship also supports the stronger association between smoking and colon cancer mortality.

The most pronounced advantage of our study was the sample size of the nationwide population-based cohort study. The number of events regarding the association between smoking and CRC mortality in our study was greater than those in previous studies [4,9,11,13,19]. We chose ever smoking as the main exposure risk because the effects of smoking may persist after changes in smoking behavior [4,39]. Studies have also reported a similar CRC risk for former and current smokers [15,21]. To obviate the influence of potential confounders, we performed propensity score matching to generate the study cohort. We further adjusted for factors, such as the alcohol consumption and body mass index, because they have been closely associated with both smoking and cancer risk [5,9,10]. The results from this and other studies demonstrate that the effects of smoking in CRC were much smaller than those in cancers of the respiratory and upper gastrointestinal tracts [16]. Nevertheless, quitting smoking for at least 20 years may still significantly reduce the risk of CRC incidence and adverse outcomes, suggesting smoking as a potentially modifiable risk factor of CRC prognosis [4,9,13,18].

It might seem peculiar that patients in the highest smoking amount category did not always have an increased risk of mortality in this study, including various subgroup analyses, as did patients in the highest smoking duration category. This might be attributable to the lower thresholds of smoking duration and amount required to increase the risk of CRC mortality in our study population. Furthermore, we speculate that the differential relationship between smoking intensity or smoking duration and CRC survival may also contribute to this phenomenon, that is, a threshold relationship for smoking intensity and a dose–response relationship for smoking duration. The relatively small number of patients in the highest smoking amount category in the whole cohort and in subgroups might also be accountable. Lastly, it might be postulated that the impact of the smoking duration on the risk of CRC mortality outweighs that of smoking intensity in our study population.

The limitation of this study stemmed largely from the use of administrative databases. The self-reported and retrospective collection of information on smoking and other variables was prone to recall and reporting biases. Data regarding family history of CRC, dietary information, physical activity, CRC screening, and use of cyclooxygenase inhibitors were not comprehensively recorded. Most importantly, other measures of smoking behavior, such as age at initiation, cumulative cigarette pack years, and passive smoking, were not collected. These factors may compromise the accuracy of the analysis. Furthermore, although we performed propensity score matching and adjusted for multiple covariates associated with smoking and CRC prognosis, the possibility of residual confounding cannot be excluded. The misclassification of anatomical subsites of CRC may have occurred, especially for tumors located in the junction of the sigmoid colon and the rectum. Finally, data regarding the molecular derangements of cancer were not available. These factors preclude a further detailed analysis of the differential effects of smoking on subsite CRC mortality.

## 5. Conclusions

Our study demonstrated that cigarette smoking was associated with a significantly, though moderately, increased risk of mortality in Asian patients with CRC. The smoking status can plausibly be considered in the risk stratification of CRC, and smoking cessation can be incorporated into comprehensive treatment planning for patients with CRC.

## Figures and Tables

**Table 1:** Demographic analysis of smoking and nonsmoking patients with colorectal cancer.

	Total	Nonsmoking	Smoking	
	*N* = 18,816	*N* = 12,544	*N* = 6272	*p*
Age group, *n* (%), years				
<40	554 (2.94)	377 (3.01)	177 (2.82)	0.8870
40–50	1298 (6.90)	859 (6.85)	439 (7.00)	
50–60	3642 (19.36)	2421 (19.30)	1221 (19.47)	
60–70	6213 (33.02)	4163 (33.19)	2050 (32.68)	
≥70	7109 (37.78)	4724 (37.66)	2385 (38.03)	
Sex, *n* (%)				
Male	17,019 (90.45)	11,346 (90.45)	5673 (90.45)	1.0000
Female	1797 (9.55)	1198 (9.55)	599 (9.55)	
Drinking, *n* (%)				
No	13,821 (73.45)	10,817 (86.23)	3004 (47.90)	<0.0001
Yes	4995 (26.55)	1727 (13.77)	3268 (52.10)	
Remote area, *n* (%)				
No	18,477 (98.20)	12,334 (98.33)	6143 (97.94)	0.0628
Yes	339 (1.80)	210 (1.67)	129 (2.06)	
Cancer subsite, *n* (%)				
Colon	12,006 (63.81)	7999 (63.77)	4007 (63.89)	0.8722
Rectum	6810 (36.19)	4545 (36.23)	2265 (36.11)	
Clinical stage, *n* (%)				
I	4472 (23.77)	2986 (23.80)	1486 (23.69)	0.9532
II	3610 (19.19)	2398 (19.12)	1212 (19.32)	
III	7125 (37.87)	4742 (37.80)	2383 (37.99)	
IV	3609 (19.18)	2418 (19.28)	1191 (18.99)	
Grade, *n* (%)				
Well-differentiated	1221 (6.49)	794 (6.33)	427 (6.81)	0.1276
Moderately differentiated	15,877 (84.38)	10,638 (84.81)	5239 (83.53)	
Poorly differentiated	1562 (8.30)	1015 (8.09)	547 (8.72)	
Undifferentiated	156 (0.83)	97 (0.77)	59 (0.94)	
CCI group, *n* (%)				
0–1	14,453 (76.81)	9644 (76.88)	4809 (76.67)	0.4946
2–4	3863 (20.53)	2579 (20.56)	1284 (20.47)	
≥5	500 (2.66)	321 (2.56)	179 (2.85)	
Death, *n* (%)	5384 (28.61)	3495 (27.86)	1889 (30.12)	0.0012

**Table 2:** Cigarette smoking associated with mortality risk of colorectal cancer.

	Patients	Death	%	Crude HR (95% CI)	*p*	Adjusted HR (95% CI) ^a^	*p*
Smoking							
Never	12,544	3495	27.86	Ref.		Ref.	
Quit/Current	6272	1889	30.12	1.11 (1.05–1.19)	0.0009	1.10 (1.03–1.18)	0.0056
Smoking count							
0	12,544	3495	27.86	Ref.		Ref.	
1–10/day	1757	518	2948	1.03 (0.93–1.15)	0.5581	1.03 (0.92–1.15)	0.6435
11–20/day	3297	1008	30.57	1.17 (1.08–1.27)	0.0001	1.16 (1.07–1.26)	0.0006
>20/day	1218	363	29.80	1.08 (0.95–1.23)	0.2270	1.07 (0.94–1.22)	0.3144
Trend test						*p* = 0.3686	
Smoking years							
0	12,544	3495	27.86	Ref.		Ref.	
1–10	1248	338	27.08	1.01 (0.89–1.15)	0.8435	1.01 (0.88–1.15)	0.9426
11–30	2528	692	27.37	1.12 (1.02–1.23)	0.0184	1.11 (1.01–1.23)	0.0356
>30	2496	859	34.42	1.15 (1.06–1.26)	0.0014	1.14 (1.04–1.25)	0.0044
Trend test						*p* = 0.0474	

**^a^** Adjusted for age (continuous), sex, drinking habit, residence in remote areas, cancer subsite, cancer clinical stage, cancer grade, and CCI group.

**Table 3:** Cigarette smoking associated with mortality risk of colorectal cancer stratified by sex.

	Men					Women				
	Patients	Death	%	Adjusted HR (95%) CI) ^a^	*p*	Patients	Death	%	Adjusted HR (95%) CI) ^a^	*p*
Smoking										
Never	11,346	3213	28.32	Ref.		1198	282	23.54	Ref.	
Quit/Current	5673	1753	30.90	1.09 (1.02–1.18)	0.0156	599	136	22.70	1.26 (0.96–1.66)	0.1032
Smoking count										
0	11,346	3213	28.32	Ref.		1198	282	23.54	Ref.	
1–10/day	1484	455	30.66	1.00 (0.89–1.12)	0.9766	273	63	23.08	1.35 (0.93–1.96)	0.1093
11–20/day	3027	951	31.42	1.16 (1.06–1.26)	0.0011	270	57	21.11	1.17 (0.79–1.74)	0.4235
>20/day	1162	347	29.86	1.06 (0.93–1.22)	0.3752	56	16	28.57	1.20 (0.61–2.36)	0.5938
Trend test				*p* = 0.4186					*p* = 0.6222	
Smoking year										
0	11,346	3213	28.32	Ref.		1198	282	23.54	Ref.	
1–10	1057	286	27.06	0.96 (0.83–1.10)	0.5430	191	52	27.23	1.59 (1.03–2.45)	0.0367
11–30	2242	645	28.77	1.11 (1.01–1.24)	0.0390	286	47	16.43	1.04 (0.70–1.55)	0.8407
>30	2374	822	34.63	1.14 (1.04–1.25)	0.0076	122	37	30.33	1.28 (0.79–2.09)	0.3108
Trend test				*p* = 0.1460					*p* = 0.8621	

**^a^** Adjusted for age (continuous), drinking habit, residence in remote areas, cancer subsite, cancer clinical stage, cancer grade, and CCI group.

**Table 4:** Cigarette smoking associated with mortality risk of colorectal cancer stratified by cancer subsite.

	Colon					Rectum				
	Patients	Death	%	Adjusted HR (95% CI) ^a^	*p*	Patients	Death	%	Adjusted HR (95% CI) ^a^	*p*
Smoking										
Never	7999	2229	27.87	Ref.		4545	1266	27.85	Ref.	
Quit/Current	4007	1206	30.10	1.12 (1.03–1.22)	0.0096	2265	683	30.15	1.08 (0.95–1.22)	0.2339
Smoking count										
0	7999	2229	27.87	Ref.		4545	1266	27.85	Ref.	
1–10/day	1134	325	28.66	1.02 (0.89–1.17)	0.7660	623	193	30.98	1.03 (0.86–1.25)	0.7316
11–20/day	2117	647	30.56	1.15 (1.04–1.28)	0.0072	1180	361	30.59	1.17 (1.01–1.35)	0.0337
>20/day	756	234	30.95	1.18 (1.01–1.39)	0.0376	462	129	27.92	0.88 (0.70–1.12)	0.2961
Trend test				*p* = 0.0463					*p* = 0.7617	
Smoking year										
0 years	7999	2229	27.87	Ref.		4545	1266	27.85	Ref.	
1–10 years	823	219	26.61	0.98 (0.83–1.15)	0.8084	425	119	28.00	1.06 (0.84–1.33)	0.6220
11–30 years	1636	453	27.69	1.13 (1.00–1.28)	0.0447	892	239	26.79	1.08 (0.90–1.28)	0.4154
>30 years	1548	534	34.50	1.18 (1.05–1.32)	0.0046	948	325	34.28	1.09 (0.93–1.27)	0.3092
Trend test				*p* = 0.0889					*p* = 0.0713	

**^a^** Adjusted for age (continuous), sex, drinking habit, residence in remote areas, cancer clinical stage, cancer grade, and CCI group.

**Table 5:** Cigarette smoking associated with mortality risk of colorectal cancer stratified by age.

	Age ≤ 60					Age > 60				
	Patients	Death	%	Adjusted HR (95% CI) ^a^	*p*	Patients	Death	%	Adjusted HR (95% CI) ^a^	*p*
Smoking										
Never	4117	829	20.14	Ref.		8427	2666	31.64	Ref.	
Quit/Current	2055	445	21.65	1.07 (0.92–1.23)	0.3971	4217	1444	34.24	1.12 (1.03–1.21)	0.0061
Smoking count										
0	4117	829	20.14	Ref.		8427	2666	31.64	Ref.	
1–10/day	590	125	21.19	0.97 (0.77–1.23)	0.8078	1167	393	33.68	1.05 (0.92–1.19)	0.4655
11–20/day	1046	232	22.18	1.19 (0.99–1.42)	0.0616	2251	776	34.47	1.16 (1.05–1.27)	0.0034
>20/day	419	88	21.00	0.93 (0.71–1.21)	0.5737	799	275	34.42	1.12 (0.97–1.31)	0.1274
Trend test				*p* = 0.9888					*p* = 0.1497	
Smoking year										
0	4117	829	20.14	Ref.		8427	2666	31.64	Ref.	
1–10	525	118	22.48	0.92 (0.72–1.17)	0.4871	723	220	30.43	1.04 (0.89–1.22)	0.6071
11–30	1200	242	20.17	1.03 (0.86–1.24)	0.7198	1328	450	33.89	1.15 (1.02–1.30)	0.0190
>30	330	85	25.76	1.43 (1.10–1.87)	0.0074	2166	774	35.73	1.12 (1.02–1.24)	0.0209
Trend test				*p* = 0.2077					*p* = 0.1265	

**^a^** Adjusted for age (continuous), sex, drinking habit, residence in remote areas, cancer subsite, cancer clinical stage, cancer grade, and CCI group.

## Data Availability

The data sources were the Taiwan Nation Health Insurance Database and Taiwan Cancer Registry. The data are available with permission from the Taiwan Health and Welfare Data Science Center (https://dep.mohw.gov.tw/DOS/np-2497-113.html, accessed on 16 November 2021). Restrictions apply to the availability of these data, which were used under license for this study.
